# HPV16-miRNAs exert oncogenic effects through enhancers in human cervical cancer

**DOI:** 10.1186/s12935-024-03364-8

**Published:** 2024-05-15

**Authors:** Yunuan Wang, Xueying Wang, Yiting Liu, Yuxin He, Xiaoling Duan, Qinmei Li, Yanchun Huang, Guoxiong Xu, Qi Lu

**Affiliations:** 1 Department of Obstetrics and Gynecology, Jinshan Hospital, Fudan University, Shanghai, 201508 China; 2 Department of Obstetrics and Gynecology, Shanghai Medical College, Fudan University, Shanghai, China; 3 Shanghai Key Laboratory of Psychotic Disorders, Shanghai Clinical Research Center for Mental Health, Shanghai Institute of Traditional Chinese Medicine for Mental Health, Shanghai Mental Health Center, Shanghai Jiao Tong University School of Medicine, Shanghai, China; 4 Research Center for Clinical Medicine, Jinshan Hospital, Fudan University, Shanghai, 201508 China

**Keywords:** Cervical cancer, Enhancer, Human papillomavirus, HPV16-miRNAs, Virus-encoded microRNAs

## Abstract

**Background:**

Cervical cancer is a human papillomavirus (HPV)-related disease. HPV type 16 (HPV16), which is the predominant cause of cervical cancer, can encode miRNAs (HPV16-miRNAs). However, the role of HPV16-miRNAs in the pathogenesis of cervical cancer remains unclear.

**Methods:**

Human cervical cancer cell lines SiHa (HPV16-positive) and C33A (HPV-negative), and cervical cancer tissues were collected to investigate the expression levels of two HPV16-miRNAs (HPV16-miR-H1 and HPV16-miR-H6). The overexpression and knockdown of HPV16-miR-H1 and HPV16-miR-H6 were performed using the lentiviral vector system and miRNA inhibitors, respectively. RNA-sequencing (RNA-seq) analysis and H3K27ac chromatin immunoprecipitation and sequencing (CHIP-seq) experiments were utilized to explore the roles of HPV16-miR-H1 and HPV16-miR-H6 facilitated by enhancers. CCK8, EdU, transwell, and wound healing assays were performed to verify the effects of HPV16-miR-H1 and HPV16-miR-H6 on cell proliferation and migration.

**Results:**

HPV16-miR-H1 and HPV16-miR-H6 were highly expressed in both SiHa cells and tissue samples from HPV16-positive cervical cancer patients. RNA-seq analysis showed that HPV16-miR-H1 and HPV16-miR-H6 induced the upregulation of numerous tumor progression-associated genes. H3K27ac CHIP-seq experiments further revealed that HPV16-miR-H1 and HPV16-miR-H6 modulated the expression of critical genes by regulating their enhancer activity. The functional study demonstrated that HPV16-miR-H1 and HPV16-miR-H6 increased the migratory capacity of SiHa cells.

**Conclusions:**

Our data shed light on the role of HPV16-encoded miRNAs in cervical cancer, particularly emphasizing their involvement in the miRNA-enhancer-target gene system. This novel regulatory mechanism of HPV16-miRNAs provides new insights and approaches for the development of therapeutic strategies by targeting HPV16-positive cervical cancer.

**Supplementary Information:**

The online version contains supplementary material available at 10.1186/s12935-024-03364-8.

## Introduction

Cervical cancer is the most frequently diagnosed female genital malignancy worldwide, with 604,000 new cases and 342,000 deaths in 2020 [1]. It is a human papillomavirus (HPV)-related disease and although the majority of HPV infections could be cleaned by the host immune responses, the persistent infection with high-risk (HR) HPV may cause precancerous lesions that can progress to cancer that usually takes about 5–10 years. HPV type 16 (HPV16) is the most common HPV type contributing to 50–55% of cases [2]. HPV prophylactic vaccine, as a primary prevention strategy for cervical cancer, can effectively prevent HPV infection. However, vaccinated people still have the possibility of HPV infection. Furthermore, the vaccine is less effective in women with previous infection or precancerous lesions [3]. The mechanisms of pathogenesis caused by HPV infection remain unclear and need to improve the understanding of disease progress and recognize molecular targets with potential for prevention and treatment.

MiRNAs are small non-coding RNA consisting of approximately 22 nucleotides. The first virus-encoded miRNA was identified in the Epstein-Barr virus (EBV) in 2004 and since then, numerous viral miRNAs have been reported in the literature [4–6]. Based on sequencing of small RNA libraries established from 2 HPV16-containing cell lines (HPK IA and HPK II cells) and 10 human cervical tissues, 5 putative HPV16-encoded miRNAs (HPV16-miRNAs, including HPV16-miR-H1, HPV16-miR-H2, HPV16-miR-H3, HPV16-miR-H5, HPV16-miR-H6) are identified and validated using mirSeqNovel [7]. Among these miRNAs, HPV16-miR-1, HPV16-miR-2, and HPV16-miR-3 are identified based on phylogenetic analysis of various HPV subtypes and bioinformatics analysis of HPV sequences with ViralmiR (move below ref 8 to here) [8], a systematic method for viral miRNA identification and viral miRNA-mediated regulatory network construction using genome-wide sequence analysis. Researchers established an HPV16-miRNAs-mediated regulatory network for HPV16 infection, in which miRNAs bind to the complementary 3′-untranslated regions (UTRs) of target mRNAs, thereby suppressing the translation of mRNAs [8, 9].

Enhancers are about 50–1500 nt in length and are commonly used as transcription factor binding platforms to increase tissue-specific transcription, regardless of their location or orientation relative to the promoter [10, 11]. Histone marker H3K27ac can be applied to distinguish active enhancers from inactive/poised enhancer elements containing H3K4me1 alone [12, 13]. Previous studies have demonstrated that miRNAs could promote gene expression by enhancer activating. For example, miR-339 upregulates the expression of G protein-coupled estrogen receptor 1 (*GPER1*) in breast cancer cells by switching on the *GPER1* enhancer to repress cell proliferation [14]. Human Identical Sequences (HIS) of SARS-CoV-2 as a miRNA can regulate the expression of inflammation-related genes by targeting their enhancers to promote COVID-19 progression [15]. It remains uncertain whether HPV16-miRNAs can modulate gene expression by altering the histone modification status to exert a pivotal regulatory influence on HPV16 infection, cervical cancer development, and metastasis.

In the present study, we explored the pattern of HPV16-miRNAs during the progression of cervical cancer from the perspective of enhancers. Specifically, we screened the expression levels of HPV16-miR-H1 and HPV16-miR-H6 in cervical cancer cell lines and tissue examples. RNA-seq and CHIP-seq were performed to explore the relationship between HPV16-miRNAs and enhancers in SiHa cells, and cell function experiments to show changes in the properties of SiHa cells. Our findings contribute to a deeper understanding of HPV16-miRNA pathogenesis in cervical cancer and highlight the potential of HPV16-miR-H1 and HPV16-miR-H6 as promising targets for the treatment of cervical cancer.

## Materials and methods

### Cell culture

Human cervical cancer cell lines SiHa (HPV16-positive) and C33A (HPV-negative), and human embryonic kidney HEK293T cells were purchased from BIOWING (BIOWING APPLIED BIOTECHNOLOGY, Shanghai, China). All cell lines were authenticated by short tandem repeat (STR) profiling with routine detection of morphology and mycoplasma. Cells were cultured at 37 °C in a humidified incubator with 5% CO_2_ and grown in DMEM/High glucose medium (Cat# SH30243.01B, HyClone, Logan, UT, USA) supplemented with 10% fetal bovine serum (Cat# 10270–106, Gibco, Thermo Fisher Scientific, Waltham, MA, USA) and 1% penicillin/streptomycin (Cat# SV30010, HyClone).

### Human specimens and ethics approval

A total of 71 cervical tissues (39 HPV16-positive cervical cancer tissues, 15 other HPV-positive cervical cancer tissues, and 17 normal cervical tissues) were collected from Jinshan Hospital, Fudan University. Patients who received radiotherapy/chemotherapy before surgery or had any other cancers were excluded. All specimens were immediately stored at − 80 °C after surgery or phlebotomise. The clinical characteristics of these cervical cancer patients, including age, HPV type, International Federation of Gynecology and Obstetrics (FIGO) stage, tumor-node-metastasis (TNM) stage, and histological type are detailed in Table S1. All tissue donations and experiments were reviewed and approved by the Ethics Committee of the Jinshan Hospital, Fudan University. Written informed consent was obtained prior to the acquisition of tissue from all patients.

### Plasmids and lentivirus package

Two HPV16-encoded microRNAs (HPV16-miR-H1 and HPV16-miR-H6) were chosen to construct plasmids. Fragments of pre-HPV-miRNAs (about 80 bp) were produced by PCR and cloned into pCDH-CMV-MCS-EF1-copGFP lentiviral vector [16] at EcoR I (5') and BamH I (3') sites (Cat# C112, Vazyme Biotech Co., Ltd, Nanjing, Jiangsu, China) to generate pCDH-pre-HPV16-miRNA. Specific primers were synthesized by Sunny (Shanghai, China). Sequences of primers are listed in Table S2. HEK293T cells were co-transfected with pCDH-pre-HPV16-miRNA, pSPAX2 (RRID: Addgene_12260), and pMD2.G (RRID: Addgene_12259) plasmids at a ratio of 4:3:1.2 via the Hieff Trans^™^ Liposomal Transfection Reagent (Cat# 40802ES02, Yeasen Biotechnology, Shanghai, China). The lentivirus was harvested at 72 h after transfection and then used to infect SiHa cells. Stable cells were selected with 1 mg/ml puromycin.

### Chemical reagents and antibodies

The inhibitors for HPV-encoded microRNAs in this follow-up study and the negative control RNA duplex (NC) were purchased from RiboBio (Guangzhou, Guangdong, China). The sequences of miRNA inhibitors are listed in Table S3. The transfection of miRNA inhibitors was performed using the Hieff Trans^™^ Liposomal Transfection Reagent (Yeasen) according to the manufacturer’s instructions. The antibody used in the ChIP-seq assays was Anti-Histone H3 (acetyl K27) antibody—ChIP Grade (Cat# ab4729, Abcam, Cambridge, UK).

### RNA extraction, RT-qPCR, and RNA-seq

Total RNA was isolated by Trizol (Cat# 10296028, Invitrogen, Thermo Fisher Scientific) and then reversed transcripted using PrimeScript^™^ RT Reagent Kit with 1 μl of gDNA Eraser (Cat# RR047A, Takara, Tokyo, Japan). The concentration and fineness of RNA were evaluated by NanoDrop ND-2000 (Thermo Fisher Scientific). SYBR Green color fluorescence quantitative premixing kit (Cat# FP205, TIANGEN, Beijing, China) was used to conduct qPCR detection in the LightCycler 480 II real-time PCR (Roche, Switzerland). U6 were used as internal reference genes and the relative expression of HPV-miRNAs and genes were calculated according to the 2^−ΔΔct^ method. The sequences of all primers are listed in Table S2. Sequencing libraries were generated using a NEBNext^®^ UltraTM RNA Library Prep Kit for Illumina® (NEB, New England Biolabs, Ipswich, MA, USA) following the manufacturer’s recommendations and index codes were added to attribute sequences to each sample. The clustering of the index-coded samples was performed on a cBot Cluster Generation System using TruSeq PE Cluster Kit v3-cBot-HS (Illumia). After cluster generation, the library preparations were sequenced on an Illumina Novaseq platform (Novaseq 6000, Illumina, San Diego, CA, USA) and 150 bp paired-end reads were generated.

Quality control was performed on the raw data, which involved the removal of adapters, poly-N, and low-quality reads, as well as calculating Q20, Q30, and GC content. Then, the high-quality clean reads were aligned to a human reference genome (GRCh38; hg38) using Hisat2 v2.0.5 [17]. The reads mapped to each gene were counted using Htseq-count v2.0.2 [18]. Differential expression analysis of two conditions (three biological replicates per condition) was performed using the DESeq2 R package (v1.34.0) [19]. Genes with a P-value < 0.05 and fold change > 1.5 found by DESeq2 were considered differentially expressed.

### CHIP-seq assay

The cells were washed twice using PBS and fixed in 1% formaldehyde for 15 min at room temperature. After the formaldehyde was quenched by 0.125 M glycine solution for 10 min, the cells were harvested and resuspended in lysis buffer (10 mM HEPES pH7.9, 1.5 mM MgCl2, 0.5% NP-40, 10 mM KCl) containing protease inhibitor cocktail (Cat# 4693132001, Roche, Basel, Switzerland). Nucleus extracts were gained and dissolved in nuclear lysis buffer (50 mM Tris–HCl pH 8.1, 0.3% SDS, 10 mM EDTA, and 1 × cocktail). Following sonication, the cell extract was incubated with antibody against H3K27ac and Protein A Dynabeads (Cat# 10002D, Invitrogen) at 4 ℃ for 16 h. A Qiagen DNA purification kit (Cat# 28106, QIAGEN, Hilden, Nordrhein-Westfalen, Germany) was then used for further immunoprecipitated. The ChIP-seq library was generated using the MicroPlex Library Preparation Kit v2 (Diagenode, Liège, Belgium) according to manufacturer instructions. The ChIP DNA libraries were sequenced using an Illumina HiSeq 2500 Platform by Shanghai Personal Biotechnology Cp. Ltd (Shanghai, China).

After strict quality control, all clean reads were aligned to a human reference genome (GRCh38; hg38) using Bowtie2 v2.3.5.1 [20] with default paired-end alignment settings. The index for the reference genome was built by using “bowtie2-build” with default parameters [20]. The aligned reads were further sorted and indexed by Samtools v1.9 [21]. Peaks calling was performed by the “callpeak” procedure from MACS3 v3.0.0 [22] using default parameters (FDR < 0.05). The genes closest to the peak or within 200 kb of the peak were subsequently annotated using the ChIPseeker R package (v1.30.3) [23].

### Transwell and wound healing assays

For the transwell assay, SiHa (5 × 10^3^ /well) cells were transfected with HPV16-encoded miRNAs lentivirus or control, and the miRNA inhibitors or the negative control RNA duplex (NC) for 3 days. Cells were then detached, re-suspended completely with serum-free medium, and added to the upper chambers of the Transwell plates (Corning Incorporated, Corning, NY, USA). At the same time, a complete medium supplemented with 20% FBS was added to the lower chamber of the Transwell plate. After 48 h incubation, cells located on the bottom surface of the upper chambers were fixed with 4% paraformaldehyde for 30 min and stained with crystal violet for 30 min. Migrant cells were counted in three random visual fields using an inverted microscope (Olympus, Tokyo, Japan).

Wound healing assays were performed as follows. Cells processed as before were seeded in a 6-well plate and scratched with a 20 μL pipette tip when 90% confluency was reached. Original scratch widths were recorded, and the scratch widths of cells after 24 h were measured. The percentage of wound healing was calculated using the following formula: (original scratch width after healing)/(original scratch width) × 100%.

### CCK8 and EdU assays

Cells transfected as before were cultured in 96-well plates with 6000 cells in each well. The cell viability was assessed using the Cell Counting Kit-8 (Cat# 40203ES80, Yeasen) at 24, 48, and 72 h, respectively. All CCK8 assays were performed in triplicates.

The proliferation of cells was tested using a BeyoClick^™^ EdU Cell Proliferation Kit with Alexa Fluor 594 (Cat# C0078S, Beyotime Institute of Biotechnology, Shanghai, China). The cells were exposed to 50 μM Edu for 2 h at 37 °C following the manufacturer’s instructions. The EdU-stained and DAPI-stained cells were visualized by fluorescence microscopy (Olympus). The analysis of cell proliferation was performed using images of randomly selected fields obtained from the fluorescence microscope. We performed three repeats for each group, and three images were used to calculate the cell proliferation rate in each repeat.

### Prediction of HPV-16 encoded miRNA targets

Human target genes of HPV-16 encoded miRNAs were predicted using TargetScan custom (https://www.targetscan.org/vert_40/seedmatch.html) and Custom Prediction methods of miRDB (https://mirdb.org/custom.html).

### Statistical analysis

All values are presented as calculated means ± standard deviations (SD) with triplicated experiments unless otherwise noted. Statistical analyses between two samples were obtained by a two-tailed Student *t*-test. The one-way ANOVA followed by the Tukey test was performed for multiple comparisons. The clinicopathological parameters were analyzed using the Mann–Whitney Test or chi-square test as indicated elsewhere. Values of P < 0.05 were taken as significant. All analyses were performed with Prism 8 (GraphPad, San Diego, CA, USA).

## Results

### HPV16-miRNAs are upregulated in cervical cancer cells and tissue specimens

The miRNA coding potential of various species of HPV with a special focus on HPV-16 was analyzed [7, 9] (Table 1). Obviously, due to the different techniques between different methods, miRNA expression may produce different results, and they were not quantitatively comparable.

**Table 1:** Prediction and validation methods of HPV-16 encoded miRNA candidates [7, 9]

miRNA name	Mature sequence	Small RNAseq Read counts^a^	Prediction with mirSeqNovel^b^	Prediction with ViralmiR^c^	TaqMan qPCR^d^	DNA PCR^e^	in situ hybridization^f^
HPV16-miR-H1	AGTGTATGAGCTTAATGATAA	45	+	+	+	+	+
HPV16-miR-H2	ATGTGTAACCCAAAACGGTTTG	1203	+	+	+	+	+
HPV16-miR-H3	*CAACTGATCTCTACTGTTA*	39	+	ND^g^	NA^h^	NA	NA
HPV16-miR-H5	*GTAAAGCATAGACCATTG*	46	+	ND	NA	NA	NA
HPV16-miR-H6	*ATCAACAACAGTAACAAA*	6161	+	+	NA	NA	NA

^a^12 libraries consisted of sequenced small RNA (fragments of 18–25 nt) libraries of two HPV 16 immortalized cell lines, HPK IA and HPK II, and 10 formalin fixed paraffin embedded tissue samples from HPV-infected cervical epithelium by SOLiD 4 platform

^b^miRSeqNovel Web site (http://sourceforge.net/projects/mirseq/files)

^c^ViralmiR Web site (http://csb.cse.yzu.edu.tw/viralmir/)

^d^HPV16-miR-H1 was detected in 4 HPV-16 containing cell lines (SiHa, CaSki, HPK IA, HPK II) and 19 tissue samples. HPV16-miR-H2 was shown by TaqMan qRT-PCR in HPK IA cells and ten tissue samples. HPV16-miR-H6 failed at assay design

^e^HPV16-miR-H1 coding regions were confirmed by PCR amplification of the relevant HPV genomic regions followed by Sanger sequencing in SiHa, CaSki, HPK IA, HPK II cell lines, and 7 tissue samples. HPV16-miR-H2 was detected in SiHa, CaSki, HPK IA, HPK II cell lines, and 6 tissue samples

^f^Expression of HPV16-miR-H1 was shown in all disease tissues by in situ hybridization. Expression of HPV16-miR-H2 was shown in only one carcinoma sample

^g^ND (Not Detected)

^h^NA (Not Applicable)

**Fig. 1:**
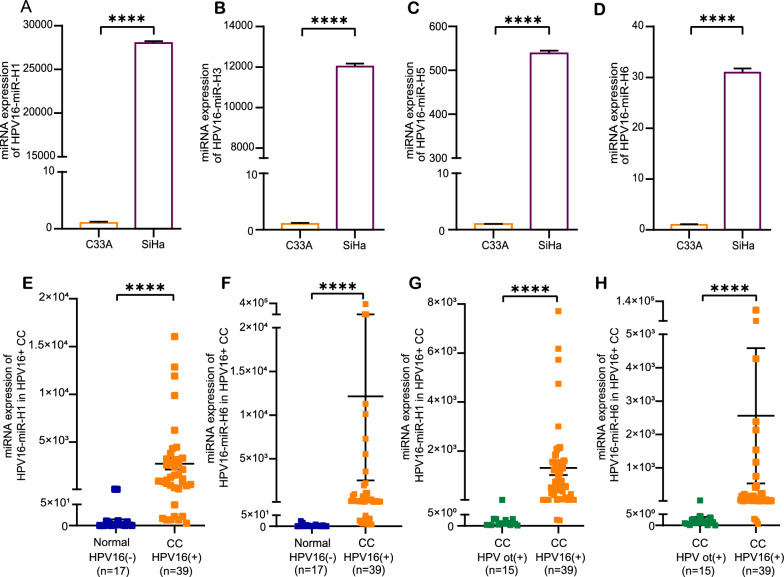
Expression of HPV16-miRNAs. **A**–**D** Expression of HPV16-miR-H1, HPV16-miR-H3, HPV16-miR-H5, and HPV16-miR-H6 in SiHa cells compared with C33A cells. **E**, **F** Expression of HPV16-miR-H1 and H6 in CC HPV 16 + (HPV16-positive cervical cancer tissue samples) compared with Normal HPV 16—(the control group). **G**, **H** Expression of HPV16-miR-H1 and H6 in CC HPV 16 + (HPV16-positive cervical cancer tissue samples) compared with CC HPV ot + (other types of HPV-positive cervical cancer samples). The data are presented as the mean ± SD. ****, p < 0.0001

In order to select representative HPV16-miRNAs to determine their specific involvement in tumorigenesis, we detected the expression of HPV16-miR-H1, HPV16-miR-H2, HPV16-miR-H3, HPV16-miR-H5, and HPV16-miR-H6 in SiHa (HPV16-positive) and C33A (HPV-negative) cervical cancer cells, respectively. Except for HPV16-miR-H2, other HPV16-miRNAs were highly expressed in SiHa, and the expression levels of HPV16-miR-H1 and HPV16-miR-H3 in SiHa cells were orders of magnitude higher than those observed in C33A cells (Fig. 1A–D; Table S4). HPV16-miR-H2 was not detectable most likely due to a problem with the primer design or low conservatism.

HPV16-miR-H1 had the highest expression level in SiHaand HPV16-miR-H6 had the highest read counts in sRNA-seq. Furthermore, HPV16-miR-H1 and HPV16-miR-H6 were predicted by two different references [7, 9]. Due to the above reasons, we detected the expression of HPV16-miR-H1 and HPV16-miR-H6 in 39 HPV16-positive cervical cancer tissue samples and 17 control tissues. Compared with the control group, the expression of HPV16-miR-H1 and HPV16-miR-H6 in HPV16-positive cervical cancer tissue samples was significantly increased (Fig. 1E, F; Table S5). We also collected 15 tissue samples from other types of HPV-positive cervical cancer, and the expression of HPV16-miR-H1 and HPV16-miR-H6 in these samples was still at low levels (Fig. 1G, H; Table S5). As shown in Table 2, there was no correlation between the clinicopathological features of cervical cancer and HPV16-miR-H6 expression. These data suggest that HPV16-positive cervical cancer may be most common and may be related to the mechanism of action of HPV16-miR-H1 and HPV16-miR-H6.

**Table 2:** Correlations between clinicopathological parameters and HPV16-miRNAs expression in CC

	Expression of HPV16-miR-H6		
Characteristic	CC > Normal (n = 35)	CC < Normal (n = 4)	p Value
Age (mean ± SD)	52.03 ± 9.89	57.5 ± 8.66	0.2835^a^
Histological type, n (%)			0.6668^b^
SCC	29	4	
Adenocarcinomas	2	0	
Adenosquamous carcinoma	4	0	
pT stage, n (%)			0.943^b^
1	17	2	
2	15	2	
3	2	0	
4	1	0	
pN stage, n (%)			0.2871^b^
0	21	4	
1	13	0	
2	1	0	
pM stage, n (%)			0.732^b^
0	34	4	
1	1	0	
FIGO stage, n (%)			0.474^b^
I	10	2	
II	11	2	
III	13	0	
IV	1	0	

The expression of HPV16-miRNAs in CC and Normal tissues was detected by qPCR. CC, HPV16 + cervical cancer; Normal, HPV16- normal. Compared with the maximum value of the Normal group (23.60), the average expression of HPV16-miR-H6 in CC higher than it is 13527.42, and the average expression of HPV16-miR-H6 in CC lower than it is 10.87

^a^Mann-Whitney test

^b^chi-square test

### HPV16-miR-H1 and HPV16-miR-H6 induce the upregulation of critical genes in cervical cancer

**Fig. 2:**
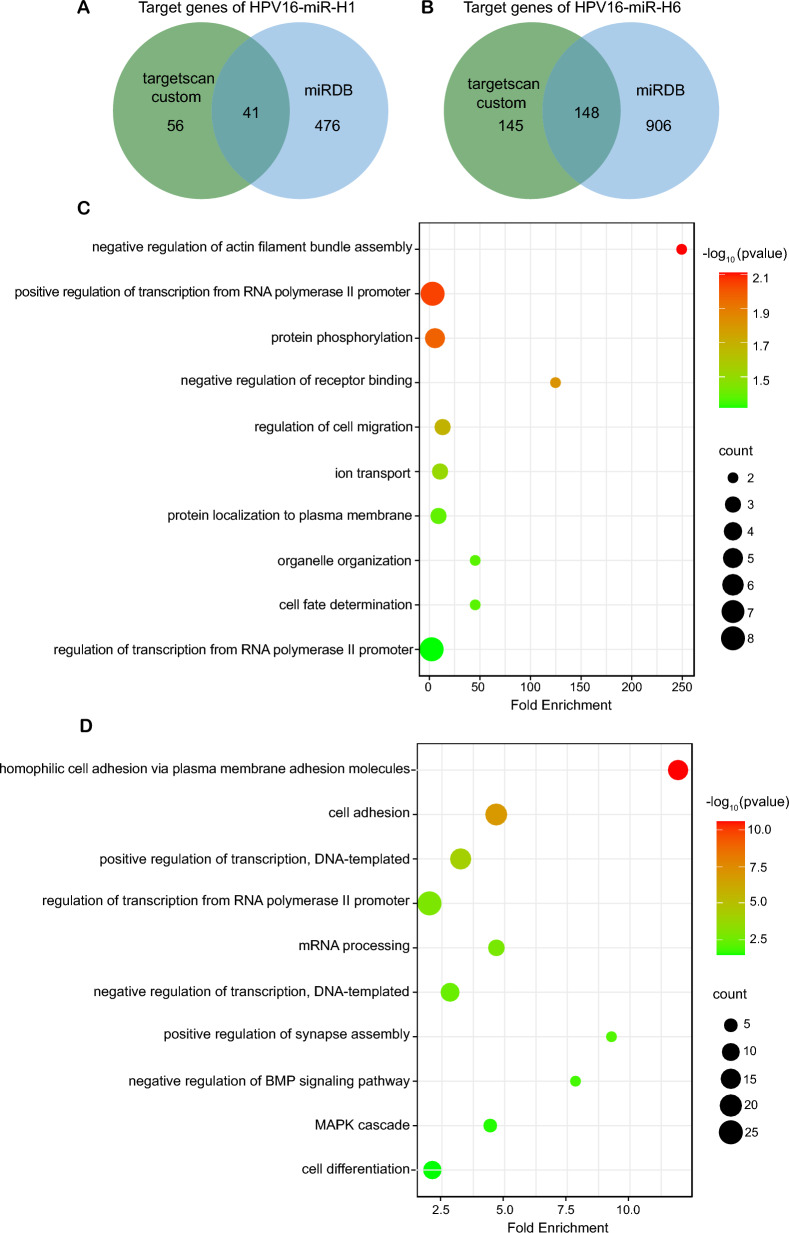
Targeted genes analysis of HPV16-miR-H1 and H6 by 3'-UTRs. **A** Genes targeted by HPV16-miR-H1 through 3'-UTRs. **B** Genes targeted by HPV16-miR-H6 through 3'-UTRs. **C** GO analysis for shared 41 genes targeted by HPV16-miR-H1 through 3'-UTRs. **D** GO analysis for shared 148 genes targeted by HPV16-miR-H6 through 3'-UTRs

Although significant progress had been achieved in the identification of HPV16-miRNAs, very little information was available on the mechanisms by which HPV16-miRNAs played a role in the promotion of cervical cancer. Since miRNA degrades mRNA through complementary binding to target gene 3'-UTRs is the classic mechanism of action of miRNA, we searched for the targets of HPV16-miR-H1 and HPV16-miR-H6 by TargetScan custom (https://www.targetscan.org/vert_40/seedmatch.html) and Custom Prediction methods of miRDB (https://mirdb.org/custom.html). In the human genome, both of these miRNAs had a unique target fingerprint of 41 and 148 shared genes respectively (Fig. 2A, B; Table S6). In particular, HPV16-miR-H1 was able to regulate actin filament bundle assembly, transcription, receptor binding, cell migration, ion transport, and cell fate (Fig. 2C). HPV16-miR-H6 targeted different genes involved in cell adhesion, transcription, mRNA processing, BMP signaling pathway, MAPK cascade, and cell differentiation (Fig. 2D).

**Fig. 3:**
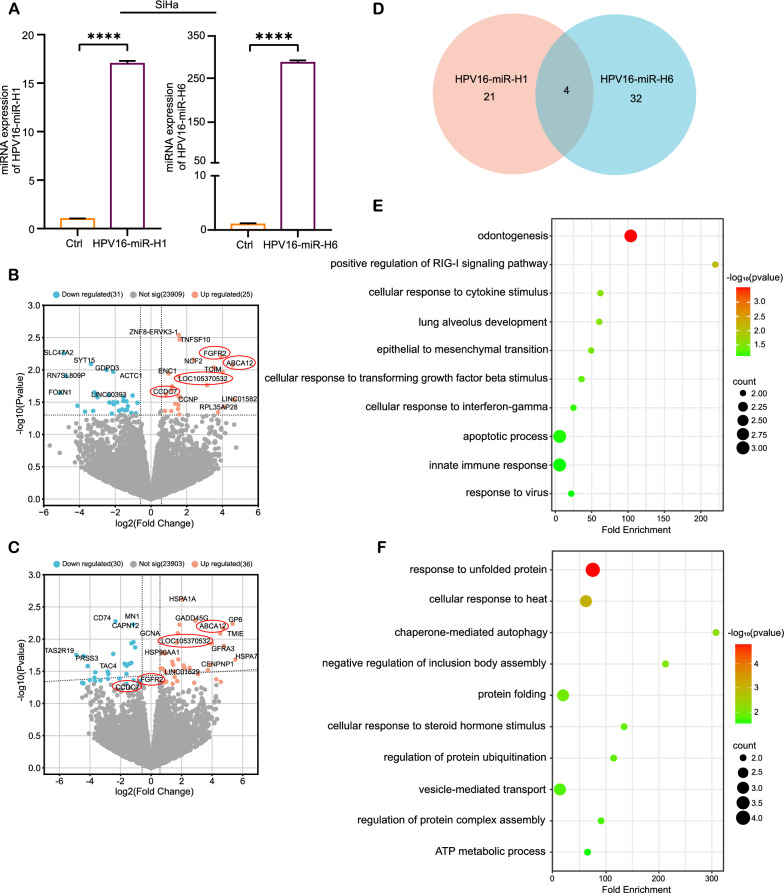
Up-regulated genes analysis by HPV16-miR-H1 and H6 in SiHa. **A** HPV16-miR-H1 and H6 were overexpressed in SiHa cells. **B**, **C** Volcano plots show the DEGs after HPV16-miR-H1 (**B**) and HPV16-miR-H6 (**C**) overexpressed in SiHa cells. **D** The Venn diagram suggests the overlapping upregulated DEGs of A and B. **E**, **F** GO analysis for upregulated DEGs by HPV16-miR-H1 (**E**) and HPV16-miR-H6 (**F**) in SiHa cells. ****p < 0.0001

In order to explore the mechanism by which HPV16-miRNAs regulate gene expression, we overexpressed HPV16-miR-H1 and HPV16-miR-H6 in the cervical cancer cell line SiHa through lentivirus infection experiments. HPV16-miR-H1 and HPV16-miR-H6 were upregulated in SiHa cells by 17- and 297-fold, respectively, compared to cells transfected with empty controls (Fig. 3A; Table S4), but due to the high background expression of HPV16-miR-H1 in SiHa, it was not possible to further upregulate its expression. The genes differentially regulated by miRNAs were analyzed by triplicate RNA-seq (fold change > 1.5, P < 0.05). We observed that HPV16-miR-H1 and HPV16-miR-H6 not only down-regulated the expression of 31 and 30 genes, respectively, but also up-regulated the expression of 25 (about 44.6%) and 36 (about 54.5%) genes, respectively (Fig. 3B–D; Table S7). The Gene Ontology (GO) analyses for these genes showed that genes upregulated by HPV16-miR-H1 were closely related to cervical cancer: positive regulation of RIG-I signaling pathway, cellular response to cytokine stimulus (including transforming growth factor beta and interferon-gamma), epithelial to mesenchymal transition, apoptotic process, innate immune response, response to the virus (Fig. 3E). Upregulated genes of HPV16-miR-H6 were associated with cellular response to unfolded protein, heat and steroid hormone, chaperone-mediated autophagy, protein folding, regulation of inclusion body and protein complex assembly, regulation of protein ubiquitination (Fig. 3F). Four genes, *FGFR2*, *ABCA12*, *CCDC7*, and *LOC105370532*, were jointly upregulated by HPV16-miR-H1 and HPV16-miR-H6. HPV16-miR-H1 and HPV16-miR-H6 may play a key regulatory role in cervical carcinogenesis by up-regulating the expression of the above host genes.

### HPV16-miR-H1 and HPV16-miR-H6 exhibit oncogenic properties via their interaction with enhancers

**Fig. 4:**
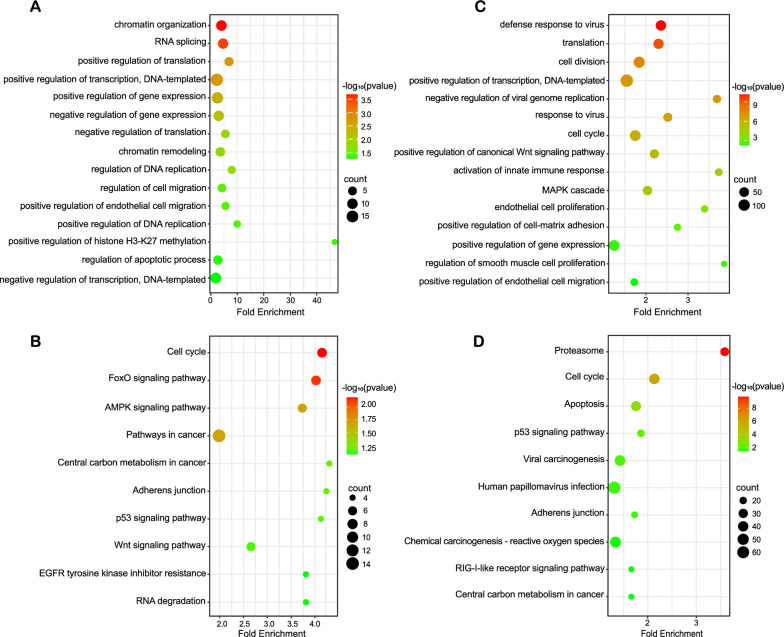
GO and KEGG analysis for peak nearest genes in SiHa cells overexpressing HPV16-miR-H1 and H6. **A**, **B** GO (**A**) and KEGG (**B**) analysis for peak nearest genes in SiHa cells overexpressing HPV16-miR-H1. **C**, **D** GO (**C**) and KEGG (**D**) analysis for peak nearest genes in SiHa cells overexpressing HPV16-miR-H6

To elucidate these abnormal regulation patterns, we focused our attention on a cis-regulatory element, the enhancers. We performed CHIP-seq of the enhancer marker H3K27ac on the cervical cancer cell line SiHa overexpressing HPV16-miR-H1, HPV16-miR-H6, and the control group and analyzed the changes of H3K27ac modification before and after miRNA overexpression. We found that overexpression of HPV16-miR-H1 in SiHa cells significantly changed the enrichment of H3K27ac modification at 303 enhancer sites (Fig. S1A, B**)**, and 2277 genes were located at 200 kb upstream and downstream of these 303 peaks. GO enrichment analysis showed that peak nearest genes mainly regulated the process of gene expression including chromatin organization, RNA splicing, DNA replication, transcription, translation, cell migration, and apoptotic (Fig. 4A), Kyoto Encyclopedia of Genes and Genomes (KEGG) enrichment showed that it was also mainly related to cancer signaling pathways, such as AMPK, P53, Wnt and cell cycle (Fig. 4B); overexpressed HPV16-miR-H6 in SiHa cells significantly changed the enrichment of H3K27ac modification at 4231 enhancer sites (Fig. S1A, C), and 16727 genes were located at 200 kb upstream and downstream of these 4231 peaks. GO enrichment analysis showed that the peak nearest genes were related to the defense response to the virus, innate immune response, cell division, proliferation, migration, adhesion, and gene expression (Fig. 4C), KEGG enrichment was related to HPV infection, viral carcinogenesis, p53, and RIG-I-like receptor signaling pathway, and cell cycle (Fig. 4D).

**Fig. 5:**
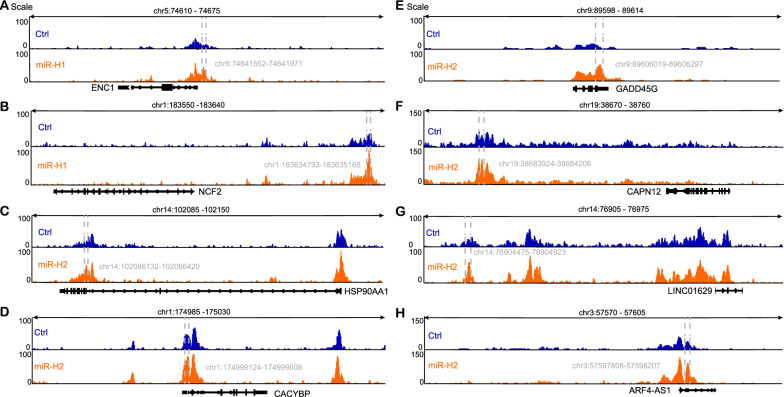
H3K27ac ChIP-seq tracks some crucial genes. **A**–**H** Between the dotted lines is the peak of H3K27ac. ENC1 (**A**). NCF2 (**B**). HSP90AA1 (**C**). CACYBP (**D**). GADD45G (**E**). CAPN12 (F). LINC01629 (**G**). ARF4-AS1 (**H**)

We further intersected the upregulated genes of HPV16-miR-H1 and HPV16-miR-H6 obtained by RNA-seq analysis with the upstream and downstream 200 kb of CHIP-seq peak of H3K27ac and obtained 2 and 18 genes, respectively. The genes upregulated by HPV16-miR-H1 through targeted enhancers include: *NCF2*, *ENC1*; the genes upregulated by HPV16-miR-H6 through targeted enhancers include: *HSP90AA1*, *LFNG*, *CACYBP*, *EEF1E1*, *GADD45G*, *DNAJB1*, *GFRA3*, *GCNA*, *CAPN12*, *DYNLT4*, *HSPA1A*, *HSPA7*, *SMIM26*, *CENPNP1*, *BMS1P4*, *LINC01629*, *SEPTIN4-AS1*, *ARF4-AS1*. Figure 5 illustrates alterations in H3K27ac modifications of selected gene enhancers**.**

### HPV16-miR-H1 and H6 enhance SiHa cell migration.

In view of the results of RNA-seq and CHIP-seq experiments, the genes activated by HPV16-miR-H1 and HPV16-miR-H6 are closely related to cancer.

**Fig. 6:**
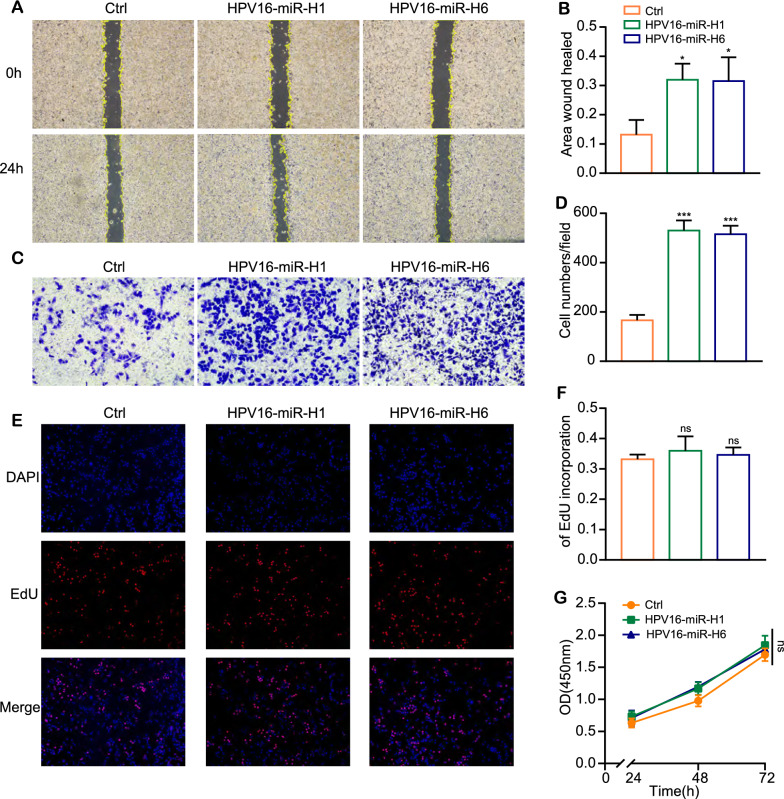
overexpression of HPV16-miR-H1 and H6 enhance migration of SiHa cells. (A-D) The effect of HPV16-miR-H1 and H6 overexpression on cell migration was detected by scratch experiments **A**, **B** and transwell assays **C**, **D**. **E**–**G** The effect of HPV16-miR-H1 and H6 overexpression on cell proliferation was detected by Edu assays **E**, **F** and CCK8 assays (**G**). CC, cervical cancer. The data are presented as the mean ± SD, ns: no significant differences, *p < 0.05, ***p < 0.001

We detected the migration ability alterations of SiHa HPV16-miRNAs groups and their corresponding controls by scratch and transwell assays. The scratch assay showed that the wound healing area of SiHa with HPV16-miRNAs groups was significantly more extensive than that of the controls at 24 h (Fig. 6A, B). For the transwell assay, 530 migrated cells were calculated in SiHa cells after transfecting HPV16-miR-H1 lentivirus, which was significantly more than the control group with 166 cells after 48 h (Fig. 6C, D). Similarly, 515 migrated cells were calculated in SiHa cells after transfecting HPV16-miR-H6 lentivirus, which was also discernibly more than the control group (Fig. 6C, D). All values were averages of three replicates. Since proliferation was another feature of cervical cancer, we performed CCK8 and EDU assays to test whether HPV16-miRNAs can promote the proliferation capacity of SiHa cells. However, the proliferation assay showed there was no significant difference in proliferation abilities between HPV16-miR-H1, HPV16-miR-H6 transfected, and control groups in SiHa cells (Fig. 6E–G). These results indicated that HPV16-miR-H1 and HPV16-miR-H6 promoted the migrating abilities of cervical cancer cells, yet had no impact on their proliferation. There was no significant difference between HPV16-miR-H1 and -H6 overexpression groups.

### HPV16-miR-H1 and H6 are promising therapeutic targets for cervical cancer

In order to explore whether HPV16-miR-H1 and HPV16-miR-H6 are potential therapeutic targets for cervical cancer, we transfected SiHa cells with inhibitor-HPV16-miR-H1, inhibitor-HPV16-miR-H6, and inhibitor-NC, performed RNA-seq and CHIP-seq of enhancer marker H3K27ac, and analyzed the differentially expressed genes and H3K27ac modification changes before and after transfection (fold change > 1.5, P < 0.05). Given that the mode of action of miRNA inhibitors is to impede miRNA function through direct binding, validating the impact of miRNA inhibitors via miRNA expression detection using RT-qPCR becomes unfeasible.

**Fig. 7:**
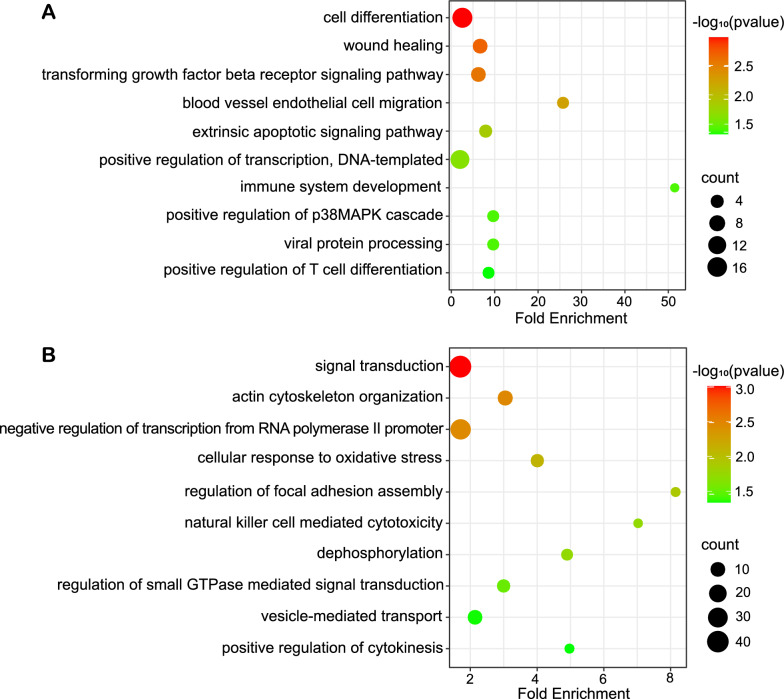
GO analysis for H3K27ac CHIP-seq in SiHa cells down-regulating HPV16-miR-H1 and H6. **A**, **B** GO analysis for genes located in the 200 kb upstream and downstream around peaks in SiHa cells transfected with inhibitor-HPV16-miR-H1 (**A**) and HPV16-miR-H6 (**B**)

Table S8 showed the differentially expressed genes of RNA-seq, some of which were closely related to cervical cancer. Transfection of inhibitor-HPV16-miR-H1 in SiHa cells significantly changed the enrichment of H3K27ac modification at 287 enhancer sites (Fig. S1D, E). There were 1791 genes located in the 200 kb upstream and downstream of these peaks, which were enriched in cell differentiation, wound healing, vascular endothelial cell migration, transcription, immunity, TGF-β receptor signaling pathway, MAPK signaling pathway, etc. (Fig. 7A). Transfection of inhibitor-HPV16-miR-H6 in SiHa cells significantly changed the degree of enrichment of the H3K27ac modification at 594 enhancer positions (Fig. S1D, F). There were 2827 genes located in the 200 kb upstream and downstream of these peaks, which were enriched in signal transduction, cell division, transcription, immunity, adhesion, dephosphorylation, etc. (Fig. 7B).

**Fig. 8:**
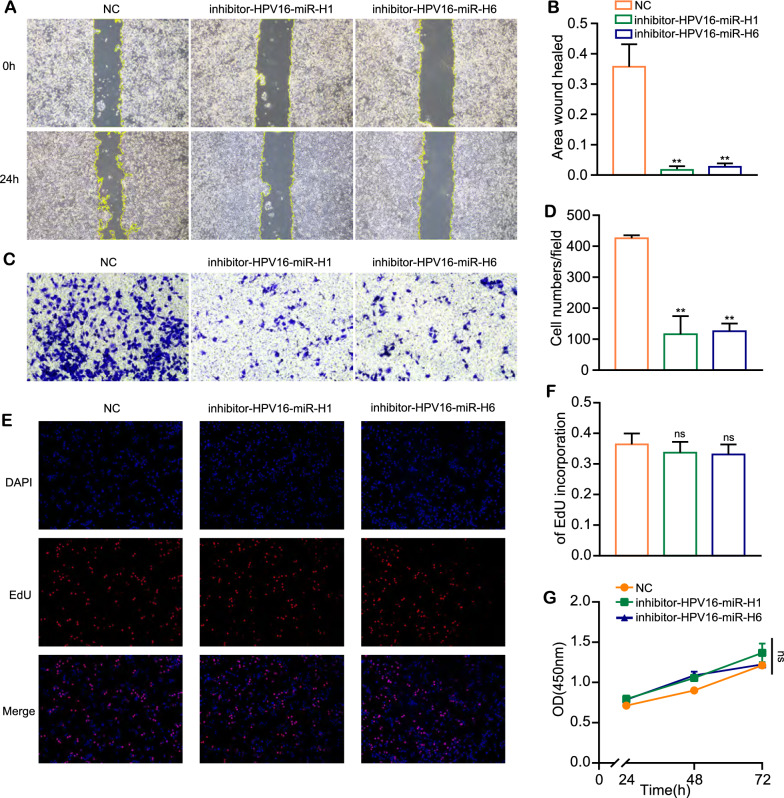
Inhibitors of HPV16-miR-H1 and H6 inhibit SiHa migration. **A**–**D** The effect of inhibitors for HPV16-miR-H1 and H6 on cell migration was detected by scratch experiments (**A**, **B**) and transwell assays (**C**, **D**). **E**–**G** The effect of inhibitors for HPV16-miR-H1 and H6 on cell proliferation was detected by Edu assays (**E**, **F**) and CCK8 assays (**G**). CC, cervical cancer. The data are presented as the mean ± SD. ns, no significant differences; **, p < 0.01

Considering that HPV16-miR-H1 and HPV16-miR-H6 can promote the migration abilities of cervical cancer cells, we wondered if inhibitors of HPV16-miRNAs play an inhibitory function on cervical cancer cells. Therefore, we detected the migration and proliferation ability alterations of SiHa cells after transfected by inhibitor-HPV16-miR-H1, inhibitor-HPV16-miR-H6, and inhibitors-NC, respectively. Cell scratch experiments showed that the wound healing area of the inhibitors transfection group was significantly smaller than that of the control group at 24 h (Fig. 8A, D). Furthermore, the group transfected with miRNA inhibitors exhibited reduced cell migration at 24 h compared to the control group in the transwell assay (Fig. 8B, E). Nevertheless, no significant difference was observed between the transfection group treated with inhibitors of HPV16-miRNAs and the control groups in CCK8 and EDU assays. (Fig. 8C, F, G). Collectively, these findings demonstrated that inhibitors targeting HPV16-miRNAs effectively suppress the migratory capacity of cervical cancer cells while not affecting their proliferation, and there was no significant difference between the two HPV16-miRNAs-blocking groups. The results revealed the inhibitory effects of these miRNA inhibitors on cervical cancer-related genes, enhancers, and cell behavior across multiple levels.

## Discussion

Cervical cancer is the most common female reproductive tract malignancy, of which repeated and persistent HR-HPV infection is the leading cause [1, 2]. Although significant progress has been made in the identification of HPV-miRNAs by bioinformatics analysis relying on HPV sequences, further studies are still required to verify the presence and elucidate the pathogenic mechanisms of HPV-miRNAs. To this end, our research combined ChIP-seq, RNA-seq, and tumor cell behavior experiments to explore the function of HPV16-miR-H1 and HPV16-miR-H6 on HPV16-positive cervical cancer cell line SiHa. We found that HPV16-miR-H1 and HPV16-miR-H6 could significantly change the gene expression, enhancer activity, and migration ability of SiHa cells. Therefore, this study advances our understanding of the role of HPV16-miR-H1 and HPV16-miR-H6 in cervical cancer.

To explore whether HPV16-miRNAs are only expressed in HPV16-infected cervical cancer cell lines, we collected SiHa (HPV16-positive cervical cancer cell line) and C33A (HPV-negative cervical cancer cell line) and found that HPV16-miR-H1, HPV16 -miR-H3, HPV16-miR-H5, and HPV16-miR-H6 were significantly higher expressed in SiHa than C33A by qPCR. We further extended our investigation to assess the expression of HPV16-miR-H1 and HPV16-miR-H6 in 71 cervical samples, and these miRNAs were elevated expressed in HPV16-positive cervical cancer samples. However, perhaps due to the difference in primers and clinical samples, based on an LNA-enhanced primer assay, previous study had shown low levels of HPV16-miRNA expression in both paraffin-embedded samples and cell lines, while no signal was detected for the presumed HPV16-miR-H2 sequence [24].

Previous reports explored the involvement of HPV16-miRNA in host cell carcinogenesis through 3'UTR: regulating critical proteins involved in immune suppression, facilitating viral immune evasion; mediating down-regulation of E6 and E7 via TEF-1, leading to increased cell cycle arrest, promoting cell cycle normalization and facilitating persistent HPV infection [7, 25]. Although miRNA can downregulate the expression of the target genes by 3'UTRs, of particular interest to us is the function of upregulated genes following overexpression of HPV16-miR-H1 and HPV16-miR-H6 in SiHa cells. GO and KEGG analysis of upregulated genes were closely related to cervical cancer: RIG-I participated in interferon production to activate innate antiviral immunity, apoptotic program, and antitumor immunity [26–29]; transforming growth factor beta (TGF-β1) functioned as a tumor inhibitor in precancerous lesions and early-stage cancers of cervix whereas as a tumor promoter in later stage [30, 31]; the epithelial to mesenchymal transition (EMT) played an important role in cervical cancer progression and metastasis [32]. Two overlapped upregulated genes by HPV16-miR-H1 and HPV16-miR-H6 have been proven to play important roles in cervical cancer: *FGFR2* inhibited cervical cancer cell viability and invasion [33], *CCDC7* activated interleukin-6 and vascular endothelial growth factor to promote proliferation via the JAK-STAT3 pathway in cervical cancer cells [34]. This suggested that the overlooked upregulated genes of HPV16-miR-H1 and HPV16-miR-H6 in SiHa cells may hold more significant roles compared to the genes downregulated by 3'UTRs.

The up-regulation of gene expression by miRNAs belongs to the noncanonical regulatory mechanism of miRNAs on gene expression. The role of miRNAs in regulating gene expression through super-enhancers has been validated by multiple studies [14, 15, 35, 36]. Consequently, we conducted H3K27ac CHIP-seq analysis in SiHa cells overexpressing HPV16-miR-H1 and HPV16-miR-H6, as well as in the control group. Our observations indicated that the overexpression of HPV16-miR-H1 and HPV16-miR-H6 resulted in the upregulation of numerous enhancers' activity. Nearest genes of enhanced enhancer by HPV16-miR-H1 were associated with chromatin organization, RNA splicing, DNA replication, transcription, translation, cell migration, apoptotic; cancer signaling pathways, such as AMPK, P53, Wnt, and cell cycle. Nearest genes of enhanced enhancer by HPV16-miR-H6 were associated with defense response to the virus, innate immune response, cell division, proliferation, migration, adhesion, gene expression; signaling pathways, such as HPV infection, viral carcinogenesis, p53 and RIG-I-like receptor signaling pathway, and cell cycle. ENC1, overlapping genes from CHIP-seq and RNA-seq, accelerated cervical cancer development by activating the ERK/MEK pathway [37]. Cell function experiments suggested that, although HPV16-miR-H1 and HPV16-miR-H6 did not affect SiHa cell proliferation, overexpression of HPV16-miR-H1 and HPV16-miR-H6 significantly facilitated the migration of SiHa cells. It had been reported that the insertion site of HPV16 in SiHa cells is 13q22, but the overlapping genes of CHIP-seq and RNA-seq are not on chromosome 13, so HPV16-miR-H1 and HPV16-miR-H6 may play the role of activating enhancers through the three-dimensional structure of the genome, which may be a general rule of the role of HPV16-miRNAs in cervical cancer [10, 38].

For patients with HPV16, we envision development therapy targeting HPV16-miR-H1 and HPV16-miR-H6 to combat cervical cancer progression. Through transfecting inhibitors of HPV16-miR-H1 and HPV16-miR-H6 into SiHa cells, we conducted RNA-seq and CHIP-seq, and observed cell behavior. RNAseq revealed some key genes in cervical cancer: the methylation level of *POU4F3* can be used as a marker of cervical cancer [39]; hypermethylation of *IGF2* is associated with a high risk of cervical cancer [40]; lncRNA TTN-AS1 participated in the development of cervical cancer by regulating the miR-573-E2F3 axis [41]. 200 kb upstream and downstream genes of weakened enhancer by HPV16-miR-H1 were associated with cell differentiation, wound healing, vascular endothelial cell migration, transcription, immunity, TGF-β receptor signaling pathway, MAPK signaling pathway; 200 kb upstream and downstream genes of weakened enhancer by HPV16-miR-H6 were associated with signal transduction, cell division, transcription, immunity, adhesion, and dephosphorylation. Cell behavior experiments show that inhibitors of HPV16-miR-H1 and HPV16-miR-H6 can inhibit the migration function of SiHa cells. Given that blocking HPV16-miR-H1 and HPV16-miR-H6 by miRNA inhibitors effectively curtails cervical cancer progression by various levels, including gene expression, histone modifications, and cell behavior, these miRNA inhibitors (inhibitor-HPV16-miR-H1 and inhibitor-HPV16-miR-H6) hold potential as novel therapeutic agents for cervical cancer.

Compared to the previous study [7, 9]*,* our data provided more samples to prove that HPV16-miR-H1 and HPV16-miR-H6 were highly expressed in both SiHa cells (HPV16-positive) and cervical cancer tissues (HPV16-positive); our study offered novel insights into the mechanism of enhancer activity of HPV16-miR-H1 and HPV16-miR-H6 in cervical cancer; and our study used functional experiments to demonstrate that HPV16-miR-H1 and HPV16-miR-H6 increase the migratory capacity of SiHa cells, which is an important feature of cancer cells.

There is still ample room for in-depth exploration in our subsequent research. Firstly, HPV16-miR-H3 and HPV16-miR-H5 are currently pending experimental investigations to elucidate their mechanism of action; Secondly, further investigations are warranted to unveil novel HPV-miRNAs and their impact on HPV infection and carcinogenesis; Thirdly, the precise genes targeted by HPV16-miR-H1 and HPV16-miR-H6 through enhancers require further in-depth investigation; Fourthly, the available studies suggested that HPV DNA can be detected in the peripheral blood samples of HPV-affected women, it is possible to investigate whether HPV infection can be determined by measuring HPV16-miRNAs expression levels in plasma samples.

## Conclusion

In conclusion, our data provides evidence to support that HPV16-miR-H1 and HPV16-miR-H6, which are highly expressed in HPV16-positive cervical cancer cell line SiHa and cervical cancer tissues, are associated with cell migration. Our study offers novel insights into the mechanism of enhancer activity of HPV16-miR-H1 and HPV16-miR-H6 in cervical cancer and highlights a promising new target for the diagnosis and treatment of cervical cancer patients.

### Supplementary Information

Additional file 1: Figure S1. H3K27ac CHIP-seq peaks distribution. (A) H3K27ac CHIP-seq peaks over chromosomes in SiHa cells overexpressing HPV16-miR-H1 and HPV16-miR-H6 respectively. (B, C) The distribution of peaks on genomic elements in SiHa cells overexpressing HPV16-miR-H1 (B) and HPV16-miR-H6 (C). (D) H3K27ac CHIP-seq peaks over chromosomes in SiHa cells down-regulating HPV16-miR-H1 and HPV16-miR-H6 respectively. (E, F) The distribution of peaks on genomic elements in SiHa cells transfected with inhibitor-HPV16-miR-H1 (F) and inhibitor-HPV16-miR-H6 (F).

Additional file 2. Supplementary Tables S1–S8.

## Data Availability

The other datasets supporting the conclusions of this article are included within the article and its additional files. Further information and requests for resources and reagents should be directed to and will be fulfilled by the corresponding author, Qi Lu (Dr_Luqi@163.com).

## Abbreviations

HPV: Human papillomavirus
EBV: Epstein-Barr virus
HR: High-risk
HPV16: Human papillomavirus type 16
HPV16-miRNAs: Human papillomavirus type 16 encoded miRNAs
RNA-seq: RNA-sequencing
CHIP-seq: Chromatin immunoprecipitation and sequencing
CCK8: Cell counting kit-8
EDU: 5-Ethynyl-2’-deoxyuridine
H3K27ac: Histone H3 Lysine 27 acetylation
UTR: Untranslated region
*GPER1*: G protein-coupled estrogen receptor 1
HIS: Human Identical Sequences
SARS-CoV-2: Severe Acute Respiratory Syndrome Coronavirus 2
COVID-19: Corona Virus Disease 2019
STR: Short tandem repeat
FIGO: International Federation of Gynecology and Obstetrics
TNM: Tumor-node-metastasis
NC: Negative control
FDR: False discovery rate
GO: Gene Ontology
KEGG: Kyoto Encyclopedia of Genes and Genomes
