# Supplementary figures and images for: HPV16-miRNAs exert oncogenic effects through enhancers in human cervical cancer
Source: Cancer Cell Int. 2024 May 15;24:172. doi: 10.1186/s12935-024-03364-8 (PMC11097496; doi:10.1186/s12935-024-03364-8)

**A**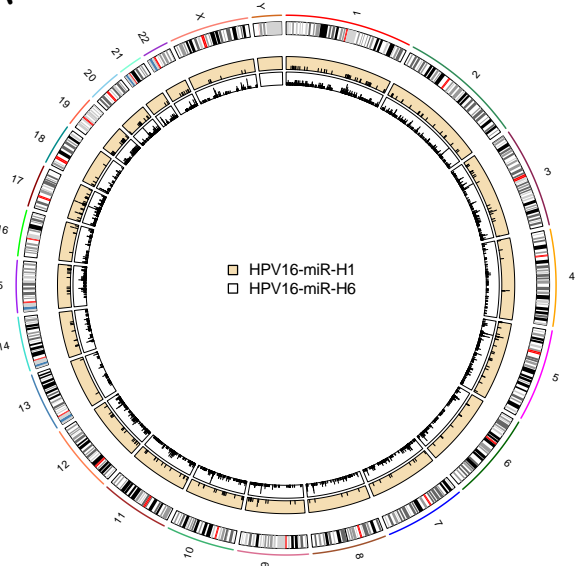**D**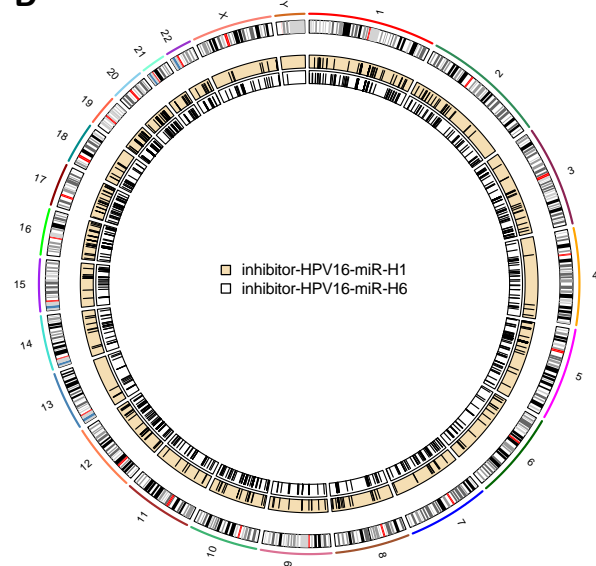**B**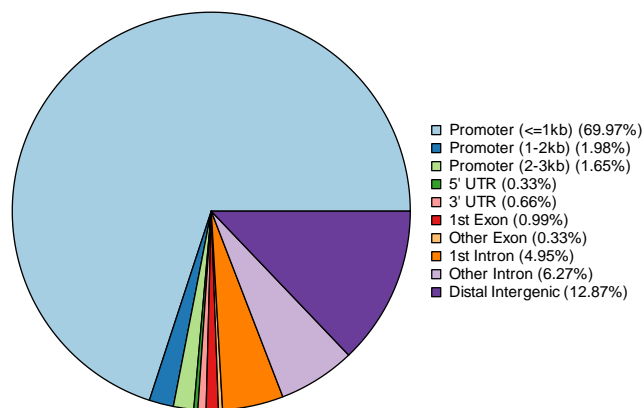**E**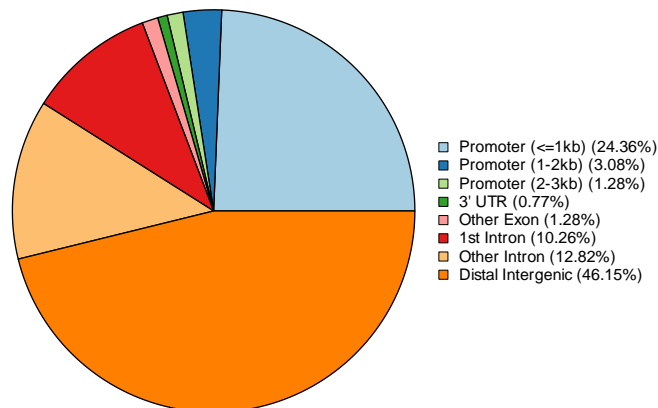**C**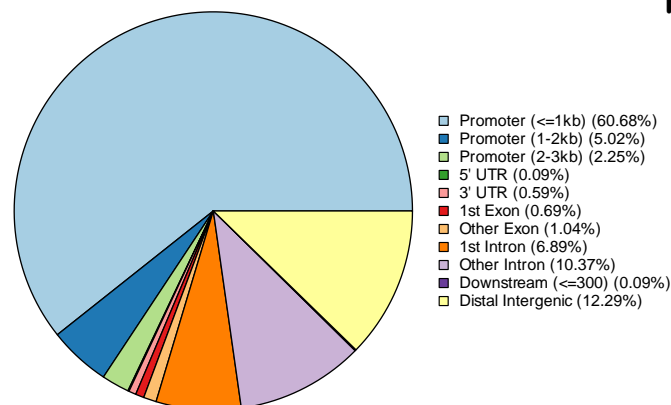**F**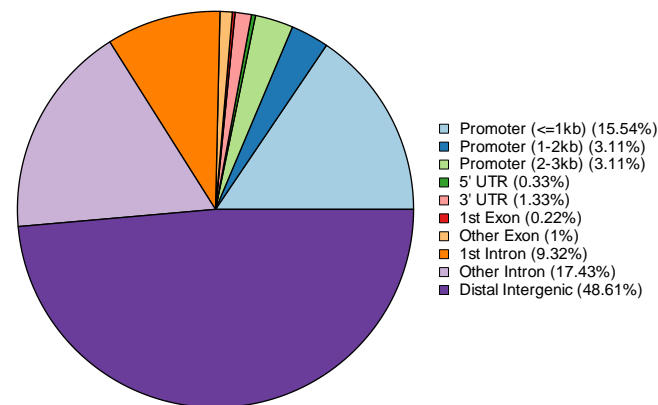

Supplement: Supplementary file 1 — Additional file 1: Figure S1. H3K27ac CHIP-seq peaks distribution. (A) H3K27ac CHIP-seq peaks over chromosomes in SiHa cells overexpressing HPV16-miR-H1 and HPV16-miR-H6 respectively. (B, C) The distribution of peaks on genomic elements in SiHa cells overexpressing HPV16-miR-H1 (B) and HPV16-miR-H6 (C). (D) H3K27ac CHIP-seq peaks over chromosomes in SiHa cells down-regulating HPV16-miR-H1 and HPV16-miR-H6 respectively. (E, F) The distribution of peaks on genomic elements in SiHa cells transfected with inhibitor-HPV16-miR-H1 (F) and inhibitor-HPV16-miR-H6 (F). [file 12935_2024_3364_MOESM1_ESM.pdf]
